# EphrinA3 is a key regulator of malignant behaviors and a potential prognostic factor in lung adenocarcinoma

**DOI:** 10.1002/cam4.4987

**Published:** 2022-06-30

**Authors:** Ruzetuoheti Yiminniyaze, Xiujuan Zhang, Ning Zhu, Jing Wang, Chengwei Li, Gulinuer Wumaier, Daibing Zhou, Jing Li, Jingwen Xia, Youzhi Zhang, Liang Dong, Yuanyuan Zhang, Shengqing Li

**Affiliations:** ^1^ Department of Pulmonary and Critical Care Medicine, Huashan Hospital Fudan University Shanghai China

**Keywords:** cancer biology, ephrinA3, lung adenocarcinoma, PI3K/Akt signaling

## Abstract

**Background:**

As a member of the Ephrin protein family that elicits short distance cell‐cell signaling, EphrinA3 has been shown to promote or inhibit tumorigenesis depending on tumor types, but its roles and the underlying mechanisms in lung adenocarcinoma (LUAD) have not been reported.

**Materials and Methods:**

The TCGA database and Kaplan‐Meier Plotter database were used to analyze the differential expression of EphrinA3 between LUAD and para‐carcinoma tissues, and its effect on overall survival of LUAD patients. CCK‐8 assay, Edu assay, and flow cytometry were used to probe the effect of EphrinA3 on the proliferation of LUAD cells, and transwell assay was employed to examine its effect on migration and invasion. In addition, the effect of EphrinA3 on the growth of LUAD was further evaluated using a xenograft tumor model.

**Results:**

EphrinA3 was expressed highly in LUAD, and its expression level was negatively correlated with the prognosis of LUAD patients. In addition, EphrinA3 promoted proliferation, migration, and invasion of LUAD cells, and accelerated tumor growth in a xenograft LUAD model. The reported EphrinA3 receptors, EphA1 and EphA10, were expressed in clinical LUAD tissues and co‐localized with EphrinA3 in LUAD cells. Mechanistically, EphrinA3/Eph signaling activated AKT, ERK, and p38MAPK, induced epithelial‐mesenchymal transition (EMT), and upregulated matrix metalloproteases‐2 and ‐9 (MMP‐2/−9).

**Conclusion:**

EphrinA3 expression was negatively correlated with prognosis of patients with LUAD. EphrinA3 promoted proliferation, migration, and invasion of LUAD cells. EphrinA3 enhanced the phosphorylation of ERK and AKT, and potentiates EMT and MMP expression in LUAD cells.

## Graphical abstract

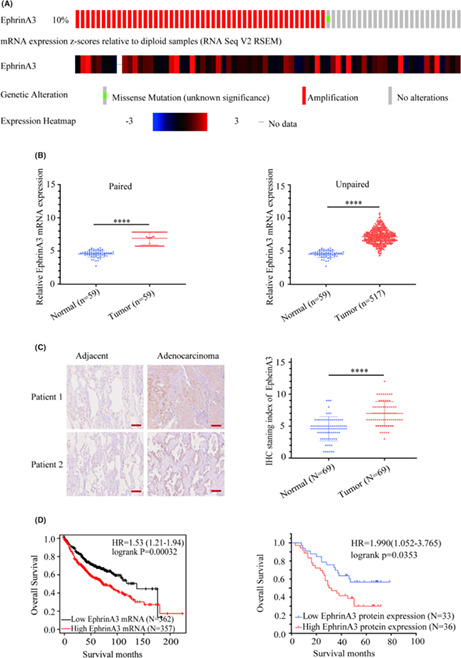

## 1 INTRODUCTION

Lung cancer is among malignancies with the highest morbidity and mortality worldwide.^1^, ^2^ Lung adenocarcinoma (LUAD) is the most common pathological type of lung cancer. The signaling network that regulates the pathogenesis of LUAD still remains to be fully elucidated. The mutations, fusions or copy number changes of a variety of genes and abnormal activation or suppression of key signaling pathways could drive the occurrence and metastasis of LUAD. These necessarily include mutations of TP53, EGFR, KRAS, fusions of ALK, ROS1, and RET, copy number changes of EGFR, KRAS, ERBB2, and aberrant activation of RTK/RAS/RAF and mTOR pathways.^3^ Despite the rapid development of targeted therapy and immunotherapy, the overall survival rate of LUAD patients is still very low due to limited responses or frequently occurring resistance to these therapeutics, underscoring the need to further identify novel key players and their crosstalk with canonical oncogenic pathways in the progression of LUAD.

Receptor tyrosine kinases (RTKs) are the second largest cell membrane receptor family, including 20 subfamilies, which are widely involved in the regulation of cell proliferation, differentiation, metabolism, survival, migration, and cell cycle. The deregulation of these RTKs and downstream signaling pathways is closely related to tumors, diabetes, severe skeletal diseases, inflammation, arteriosclerosis, and angiogenesis.^4^, ^5^ Eph is the largest subfamily of the RTKs family, and upon engaged by their cognate ligands, Ephrins, could signal bidirectionally to regulate cell rejection, adhesion, proliferation, migration, and axon formation.^6^, ^7^

In our previous studies, candidate genes in Eph family were screened according to (1) their differential expression between LUAD and paracancerous according to GEPIA database (Figure S1), and (2) their association with prognosis in LUAD according to Kaplan‐Meier Plotter database (Table S1). Finally, EphrinA3 was selected for further investigation of a potential role and molecular mechanism in LUAD.

EphrinA3, encoded by *EFNA3* on chromosome 1q21.3, is a protein with 238 amino acids (molecular weight 26.35 KDa), located predominantly on the cytomembrane. EphrinA3 and its EphA subfamily receptors are critically involved in the development and metastasis of a variety of tumors. However, EphrinA3/EphA plays distinct roles in different malignancies. EphrinA3 functions as a tumor suppressor in malignant peripheral nerve sheath tumor and oral and contributes to carcinogenesis in liver cancer.^8^, ^9^ This study aimed to elucidate the role and molecular mechanism of EphrinA3 in LUAD.

## 2 MATERIALS AND METHODS

### 2.1 Data source

The cBioportal for Cancer Genomics were used to analyze *EFNA3* genetic alteration frequency in LUAD. Gene Expression Profiling Interactive Analysis (GEPIA) was used to analyze Eph family members mRNA expression level, while the correlation of Eph family members mRNA level and overall survival in LUAD was clarified by Kaplan‐Meier Plotter database. In addition, a total of 576 samples were collected from The Cancer Genome Atlas (TCGA) database, including 517 LUAD and 59 pericarcinomatous normal tissues. Furthermore, 69 patients with LUAD were enrolled for immunohistochemistry to verify EphrinA3 differential expression in LUAD and paracancer and the relationship between EphrinA3 protein levels and overall survival. The relationship between EphrinA3 expression and gender, age, pathological grade, and TNM stage of the enrolled patients was shown in Table S2.

### 2.2 Cell lines and cell culture

BEAS‐2B and A549 cells were cultured in DMEM supplemented with 10% fetal bovine serum (Gibco), 1% penicillin (Gibco), and streptomycin (Gibco). H1299, H1975, and PC‐9 cell lines were cultured in RPMI‐1640 medium supplemented with 10% FBS (Gibco), 1% penicillin and streptomycin. All of the above cells were maintained in a 5% CO_2_ incubator at 37°C.

### 2.3 Cell counting kit‐8 assay (CCK‐8)

2 × 10^3^ cells/well with 100 μl culture medium were seeded into each 96‐well plate. After culturing for 4 h, 110 μl complete culture medium containing 10 μl CCK‐8 reagent (Yeasen) was added to respective wells at 0, 24, 48 and 72 h. The absorbance value was measured by microplate reader after incubation of the plates in 37°C for 3 h. Independent experiments were repeated for three times.

### 2.4 EDU assay

Cells with exponential growth in six‐well plates were added with 2 × EDU (Beyotime) working solution and incubated at 37°C for 2 h. The cells were fixed with 4% paraformaldehyde at room temperature for 15 min and then washed with a washing solution (PBS solution containing 3% BSA) for three times. Then the cells were added permeable solution (PBS containing 3%Triton X‐100) and incubated at room temperature for 15 min. After removing permeable solution and washing with washing solution for three times, the cells were added click reaction (500 μl/well) and incubated for 30 min at room temperature in dark. After washing three times, the cells were incubated with 1 × Hoechst 33342 solution at room temperature for 10 min for nuclear staining. Finally, the cells were washed three times and photographed using a fluorescence microscope for three random fields/wells.

### 2.5 Flow cytometry for cell cycle

After being digested and centrifuged, the exponentially growing cells were resuspended with 75% ethanol precooled at −20°C and placed at −20°C overnight. The next day, cells were centrifuged and washed twice with PBS, and then incubated in PI working solution [Dyeing buffer + Propiridine iodide PI staining solution (20X) + RNaseA(50X)] (Beyotime) at room temperature in dark for 30 min. Finally, the cell cycle was measured and analyzed by flow cytometry.

### 2.6 Transwell migration and invasion assay

For migration assay, 2 × 10^4^ cells in 200 μl DMEM or RPMI‐1640 medium without FBS were seeded into the upper chamber of transwell plate, and 800μL DMEM or RPMI‐1640 complete medium containing 10% FBS were placed into the lower chamber. For invasion assay, the upper chamber of transwell plate was pre‐coated with 60 μl 1:5 mixture of BD matrigel (Corning) and DMEM or RPMI‐1640 for 3 h at 37°C, and then the same number of cells and medium were inoculated into the upper and lower transwell chambers, respectively. After 20 h (for migration assay) or 24 h (for invasion assay), wells were fixed in methanol for 30 min and stained by crystal violent for 5 min. The number of cells from three random fields for each well were included in the statistics.

### 2.7 Western blot assay

Total protein of cell lines were extracted using RIPA lysis buffer supplemented with protease and phosphatase inhibitors. The protein bands were transferred onto polyvinylidene membranes for 70 min at 300 mA. Membranes were blocked with 5% defatted milk in TBST at room temperature for 1 h, and then incubated with anti‐GAPDH antibody (1:100000, Proteintech), anti‐EphrinA3 antibody (1:1000, Santa Cruz, Proteintech), anti‐p38MAPK antibody (1:1000, Abcam), anti‐p‐p38MAPK antibody(1:1000, CST), anti‐ERK antibody (1:1000, CST), anti‐p‐ERK antibody (1:2000, CST), anti‐AKT antibody (1:1000, CST), anti‐p‐AKT(Ser473) antibody (1:2000, CST), anti‐cyclinD1 antibody (1:1000, Affinity), anti‐MMP‐2 antibody (1:1000, CST) and anti‐MMP‐9 antibody (1:1000, CST), anti‐E‐cadherin antibody (1:1000, CST), anti‐N‐cadherin antibody (1:1000, CST) at 4°C overnight. The next day, the membranes were incubated with secondary antibodies for 1 h at room temperature and exposed on Automatic chemiluminescence image analysis system (Tanon 5200).

### 2.8 Immunohistochemistry method

Paraffin‐embedded tissues were deparaffinized in xylene and rehydrated in alcohol. The tissues were heated in a pressure cooker for 5 min, and were treated with 3% H_2_O_2_ for 10 min. The tissues were incubated with goat serum for 30 min, and then incubated with primary antibody at 4°C overnight. The next day, the tissues were incubated at room temperature for 30 min with secondary antibodies, followed by DAB coloring for 10 min. Finally, photographs were taken under an inverted microscope. The score was calculated based on the intensity of staining and the proportion of positive tumor cells. Staining index = staining intensity × proportion of positive cells. Staining intensity score: Negative, 0 point; weak positive, 1 point; moderate positive, 2 points; strong positive, 3 points. The score of positive cell proportion: Less than 5%, 0 point; 5%–25%, 1 point; 26%–50%, 2 points; 51 ~ 75%, 3 points; more than 75%, 4 points. When the staining was heterogeneous, each independent staining index was added.

### 2.9 Immunofluorescence assay

The cell slides was fixed with 4% cold paraformaldehyde for 20 min and permeated with 0.2%Triton X‐100 for 10 min. Subsequently, the cells of the sliver were blocked with serum for 30 min and then incubated with primary antibodies at 4°C overnight. On the next day, the cells were incubated with second antibody for 2 h at room temperature in dark. Finally, the cells were photographed under a microscope.

### 2.10 Knockdown and overexpression of EphrinA3 in lung adenocarcinoma cell lines

Recombinant lentiviruses overexpressing EphrinA3 or EphrinA3‐targeting short heparin RNAs (shRNAs) were purchased from Shanghai Chaole Biological Company. Lentivirus was mixed with polybrene and used to infect LUAD cells. Fresh medium was replaced 24 h later, and purinomycin was added to select successfully infected cells 48 h later.

### 2.11 Animal model and in vivo imaging

Six BALB/C nude mice purchased from Shanghai Slack Laboratory Animal Company were divided into two groups randomly, and fed under pathogen‐free conditions. H1299 cells overexpression negative control (NC) or EphrinA3‐targeting shRNA (sh‐EphrinA3‐1) were further infected with lentiviruses to express firefly luciferase, and 2 × 10^6^ cells were inoculated into the right axillary fossa of nude mice. Six weeks after inoculation, D‐luciferin potassium salt (15 mg/ml) was injected intraperitoneally at 10 μg/g body weight in nude mice, followed by intraperitoneal injection chloral hydrate 5 min later. After 15 min, images were performed on Lumina‐K scan, and the resected subcutaneous tumors were weighed and immunohistochemical staining.

### 2.12 Statistical analysis

*t*‐test (paired or unpaired) or one‐way ANOVA test was performed to analyze the data when appropriate. Dichotomous variables were analyzed using Pearson's χ^2^ test. Log‐rank test was carried out to analyze the relationship between EphrinA3 expression and overall survival. *p* value <0.05 was considered as statistical significant. GraphPad Prism 8 and SPSS Statistics 23 were used for statistical analysis.

## 3 RESULTS

### 3.1 High Ephrin A3 expression correlates with poor prognosis of LUAD patients

**FIGURE 1:**
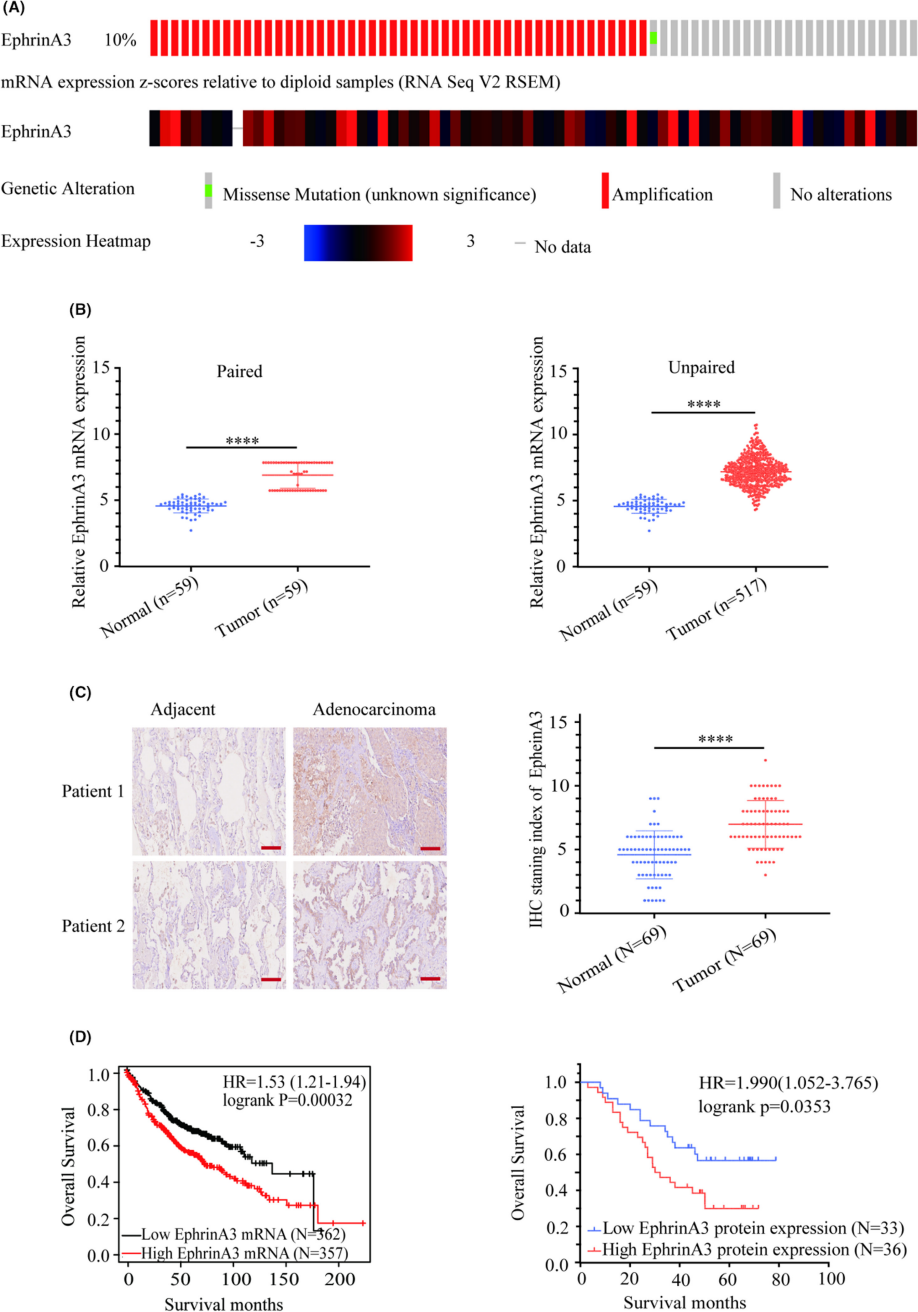
High EphrinA3 expression correlates with poor prognosis of LUAD patients. (A). Genetic alteration frequency of *EFNA3* in LUAD according to cBioportal database. (B). EphrinA3 mRNA levels were higher that adjacent normal tissues according to TCGA database. (C).EphrinA3 protein levels were higher than adjacent normal tissues according to immunohistochemical assay. Scale bar represents 100 μm; Data are mean ± SD. ****p < 0.0001; (D). EphrinA3 expression levels were negatively correlated with prognosis of LUAD patients according to Kaplan–Meier curve.

Cbioportal database showed that the genetic alteration frequency of EphrinA3 in LUAD was 10%, the majority of which was gene amplification (Figure 1A). Compared with pericarcinomatous tissues, EphrinA3 mRNA levels were significantly increased in LUAD according to the TCGA database (*p* < 0.0001) (Figure 1B). In addition, immunohistochemical results confirmed that the levels of EphrinA3 protein in LUAD were higher than those in adjacent normal tissues (*p* < 0.0001) (Figure 1C). Both the mRNA and protein levels of EphrinA3 were negatively correlated with the prognosis of LUAD patients according to Kaplan‐Meier database(Figure 1D, left, HR = 1.53; 95% CI, 1.21–1.94; *p* = 0.00032; right, HR = 1.990; 95% CI, 1.052–3.765; *p* = 0.0353). Thus, the prognostic value of EphrinA3 suggests that it plays an essential role in the pathogenesis of LUAD.

### 3.2 EphrinA3 promotes proliferation of LUAD cells

**FIGURE 2:**
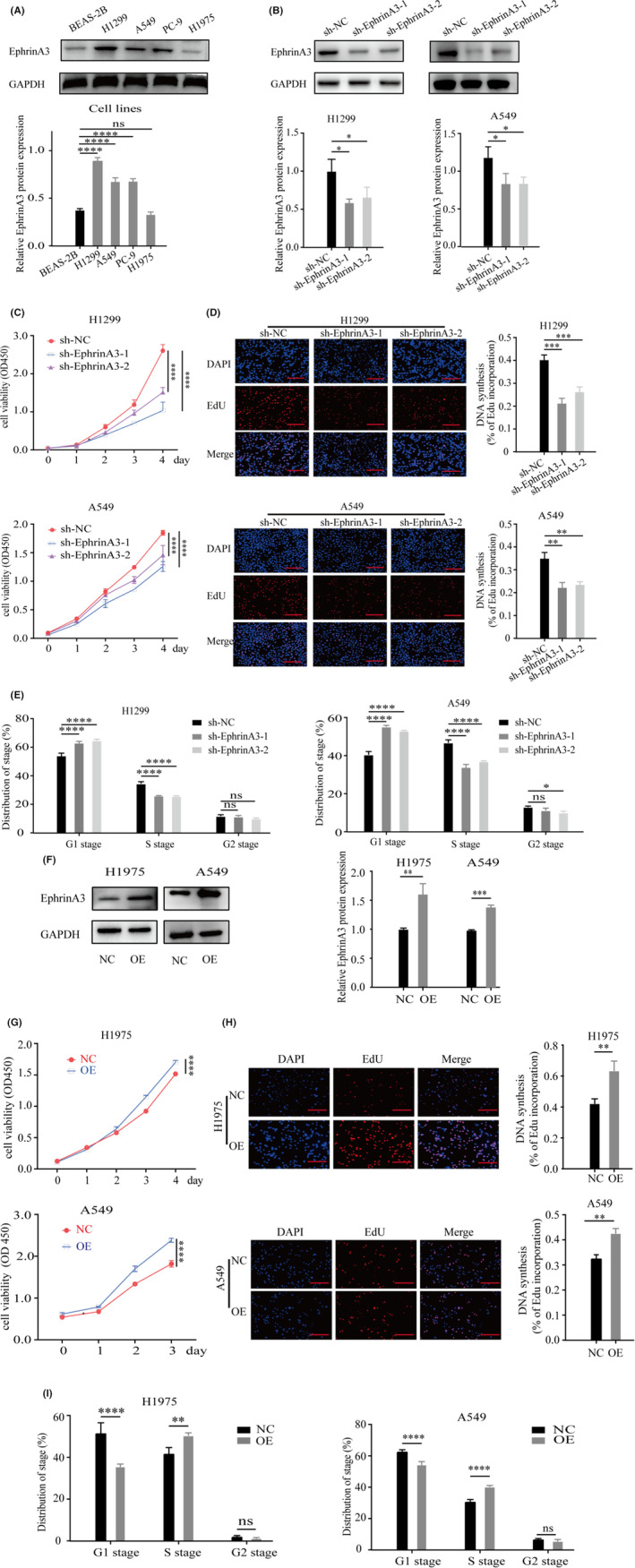
EphrinA3 promotes proliferation of LUAD cells. (A). EphrinA3 protein expression levels in BEAS‐2B and LUAD cells. Data are mean ± SD. ns: no significance, ****p < 0.0001; (B). Knockdown of EphrinA3 in H1299 and A549 cells. Data are mean ± SD. *p < 0.05; (C, D). EphrinA3 knockdown inhibits proliferation of aLUAD according to CCK‐8 and Edu assay, respectively. Scale bar represnts 200 μm; Data are mean ± SD. **p < 0.01, ***p < 0.001, ****p < 0.0001; (E). Flow cytometry assay shows that Ephrin A3 knockdown significantly increased G1 phase cells and decreased S phase cells. Data are mean ± SD. ns: no significance, *p < 0.05, ****p < 0.0001; (F). Overexpression of EphrinA3 in H1975 and A549 cells. Data are mean ± SD. **p < 0.01, ***p < 0.001; (G, H). EphrinA3 overexpression promotes proliferation of LUAD cells. Scale bar represents 200 μm; Data are mean ± SD. **p < 0.01, ****p < 0.0001; (I). Flow cytometry assay shows that Ephrin A3 overexpression significantly decreased G1 phase cells and increased S phase cells. Data are mean ± SD. ns: no significance, **p < 0.01, ****p < 0.0001.

We detected the discrepant expression of EphrinA3 in various LUAD cell lines (Figure 2A). EphrinA3 was then knocked down by shRNAs in LUAD cell lines, H1299 and A549 (Figure 2B). CCK‐8 assay showed that EphrinA3 knockdown inhibited the proliferation of LUAD cells (*p* < 0.0001) (Figure 2C), which was also confirmed by Edu assay (*p* < 0.0001) (Figure 2D). Moreover, flow cytometry assay showed that Ephrin A3 knockdown significantly increased G1 phase cells and decreased S phase cells (*p* < 0.0001) (Figure 2E), suggesting cell cycle arrest in G1 phase. Next, EphrinA3 was overexpressed in H1975 and A549 cells (Figure 2F). CCK‐8 and Edu assays both showed that EphrinA3 overexpression promoted the proliferation of LUAD cells (Figures 2G,H). Furthermore, EphrinA3 overexpression significantly decreased G1 phase cells and increased S phase cells according to flow cytometry (*p* < 0.01) (Figure 2I), indicating that it accelerated entry into S phase of the cell cycle thereby promoting cell proliferation.

**FIGURE 3:**
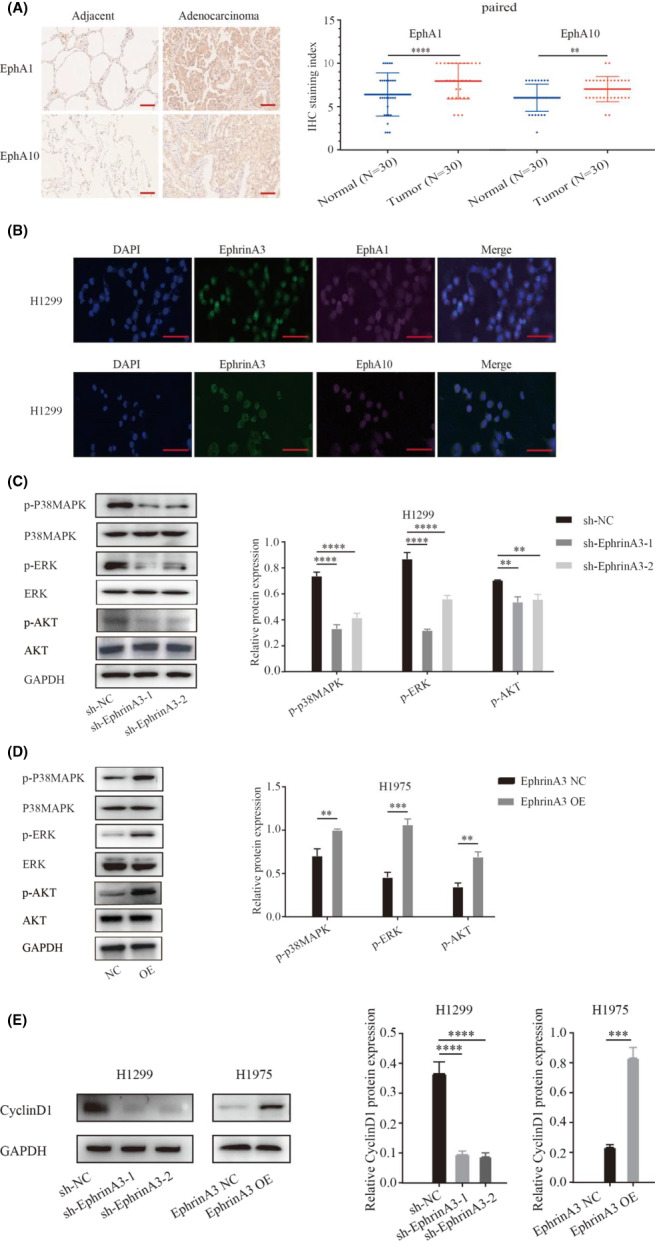
EphrinA3 activates oncogenic pathways of LUAD through interaction with tumor cells. (A). EphA1 and EphA10 protein levels were higher than adjacent normal tissues in LUAD according to immunohistochemical assay. Scale bar represents 100 μm; Data are mean ± SD. **p < 0.01, ****p < 0.0001; (B). Immunofluorescence assay shows that EphrinA3 co‐localizes with EphA1 and EphA10 in LUAD cells. Scale bar represents 100 μm; (C, D). EphrinA3 knockdown inhibits phosphorlation of Akt, ERK, and p38MAPK, while its overexpression is the opposite. Data are mean ± SD. **p < 0.01, ***p < 0.001, ****p < 0.0001; (E). EphrinA3 promotes CyclinD1 expression. Data are mean ± SD. ***p < 0.001, ****p < 0.0001.

EphrinA3 mediates cell‐cell communications through binding to EphA subfamily receptors.^10^ Consistently, EphA1 and EphA10, but not EphA8, were found substantially expressed on LUAD cells according to the TCGA database (Figure S1) and in our immunohistochemistry assay of clinical specimens (Figure 3A). Immunofluorescence assay showed that EphA1 or Eph10 colocalized with EphrinA3 on the cytomembrane of LUAD cells (Figure 3B). EphrinA3 knockdown repressed the phosphorylation of Akt, ERK, and p38MAPK, while EphrinA3 overexpression enhanced activation of these kinases (Figures 3C,D). In addition, EphrinA3 knockdown downregulated cyclin D1, a key regulator of the G1 checkpoint (Figure 3E). Together, these data suggest that Ephrin A3 signals to promote the mitosis of LUAD cells.

### 3.3 EphrinA3 promotes migration and invasion of LUAD cells

**FIGURE 4:**
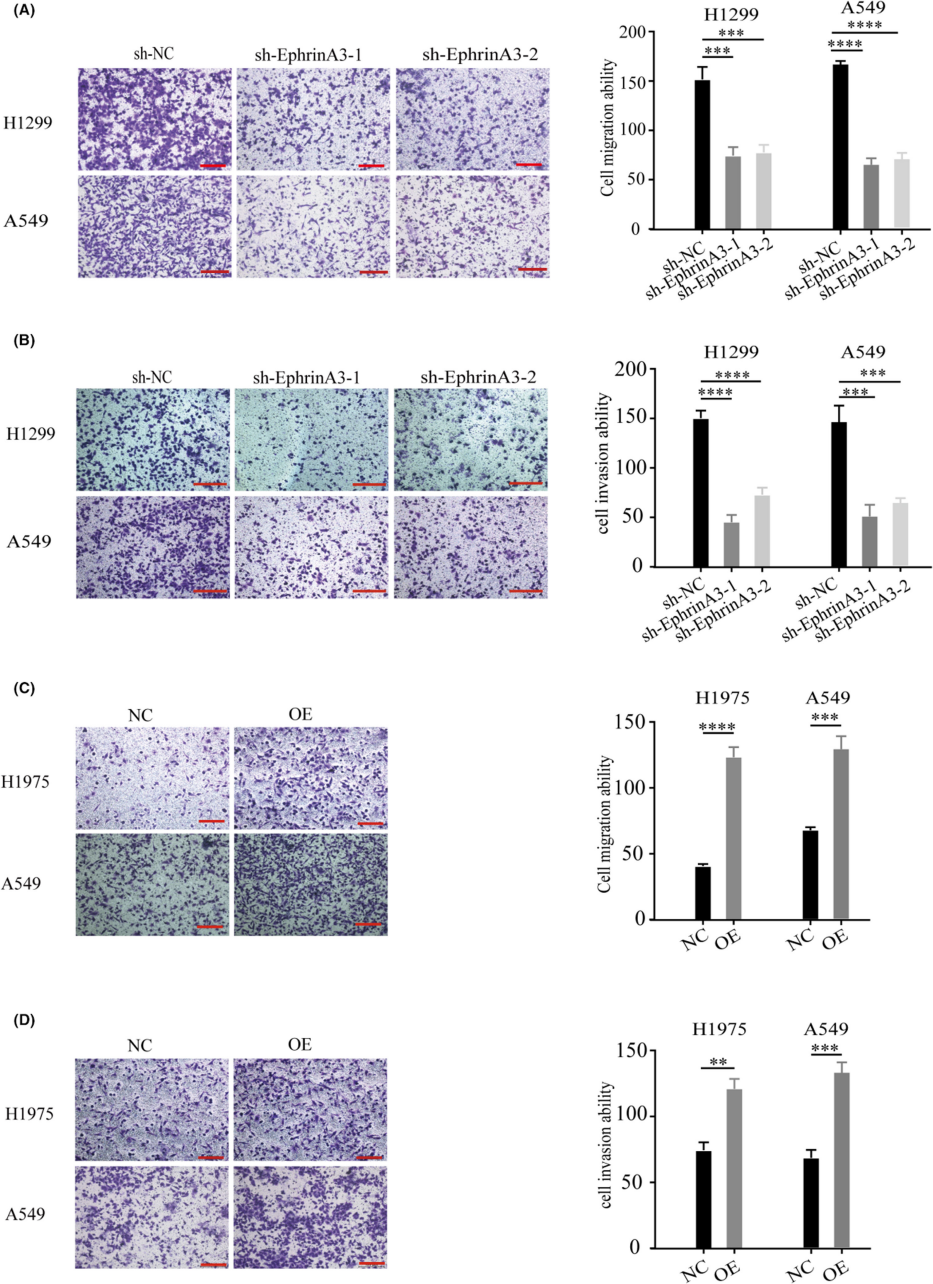
EphrinA3 promotes migration and invation of LUAD cells. (A, B). EphrinA3 knockdown inhibits migration and invasion of LUAD cells according to transwell assay. Scale bar represents 100 μm; Data are mean ± SD. ***p < 0.001, ****p < 0.0001; (C, D). EphrinA3 overexpression promotes migration and invasion of LUAD cells according to transwell assay. Scale bar represents 100 μm; Data are mean ± SD. **p < 0.01, ***p < 0.001, ****p < 0.0001.

**FIGURE 5:**
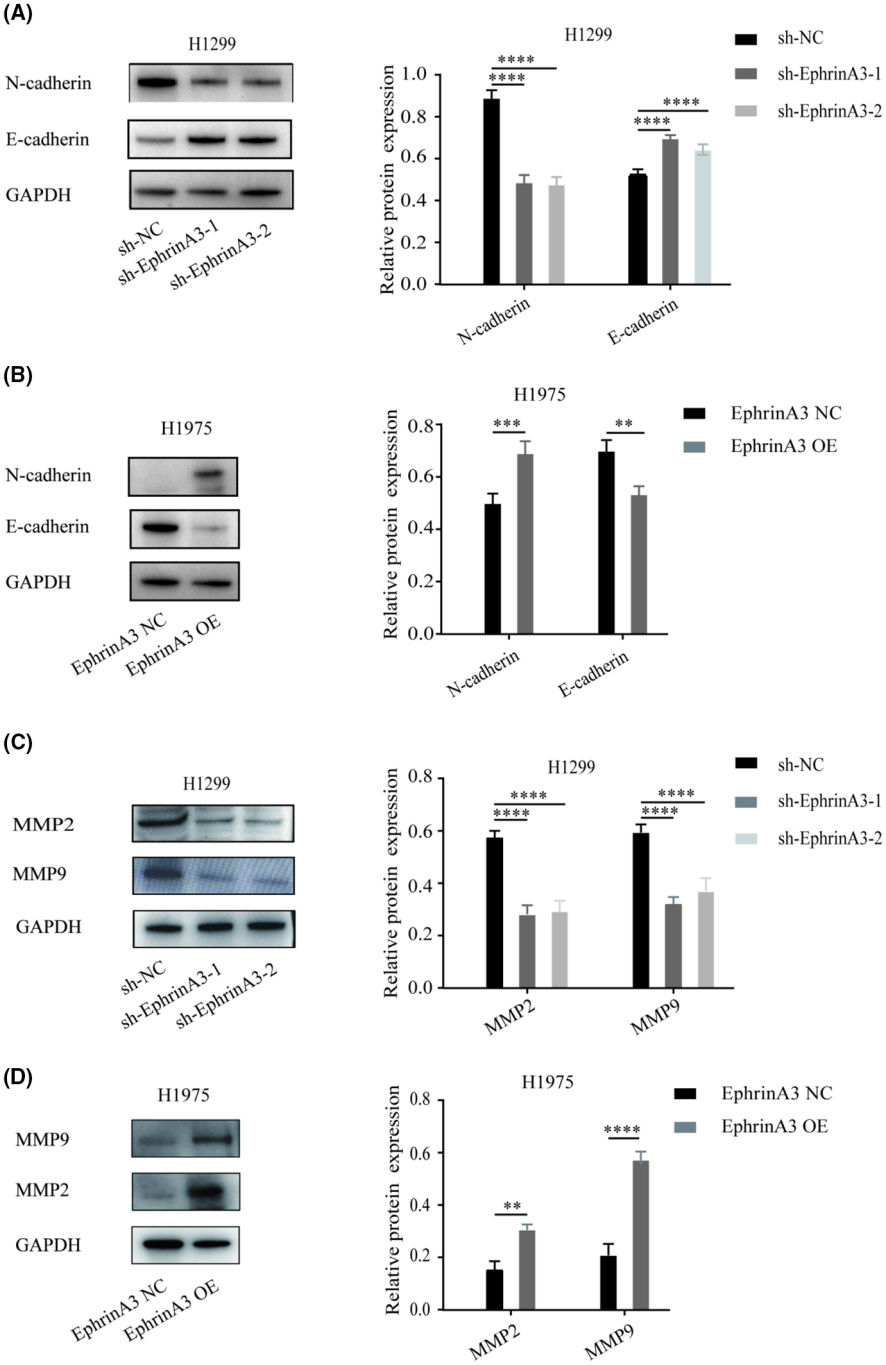
EphrinA3 promotes EMT transition and degradation of MMPs. (A, B). EphrinA3 knockdown inhibits EMT process, while its overexpression promotes EMT process. Data are mean ± SD. **p < 0.01, ***p < 0.001, ****p < 0.0001; (C, D). EphrinA3 knockdown inhibits MMP‐2 and MMP‐9 expression, while its overexpression is the opposite. Data are mean ± SD. **p < 0.01, ****p < 0.0001.

We next probed whether EphrinA3 plays a regulatory role in the migration and invasion of LUAD cells. Transwell assays showed that EphrinA3 knockdown inhibited the motility (*p* < 0.001) (Figure 4A) and invasiveness (*p* < 0.001) (Figure 4B) of H1299 and A549 cells. Conversely, EphrinA3 overexpression promoted the capabilities of migration and invasion of H1975 and A549 cells (*p* < 0.01) (Figures 4C,D). Mechanistically, we observed that EphrinA3 silencing enhanced the expression of the epithelial marker, E‐cadherin, and downregulated the messenchymal marker, N‐cadherin (Figure 5A), whereas EphrinA3 overexpression increased N‐cadherin and reduced E‐cadherin levels on LUAD cells (Figure 5B). In addition, EphrinA3 knockdown and overexpression impaired and enhanced the expression of matrix metalloproteases‐2 and ‐9 (MMP‐2/−9), respectively (Figures 5C,D). These data indicated that EphrinA3 signaling is likely to promote the migration and invasion of LUAD cells through induction of epithelial‐mesenchymal transition (EMT) and degradation of extracellular matrix.

### 3.4 EphrinA3 potentiates LUAD growth in a murine xenograft model

**FIGURE 6:**
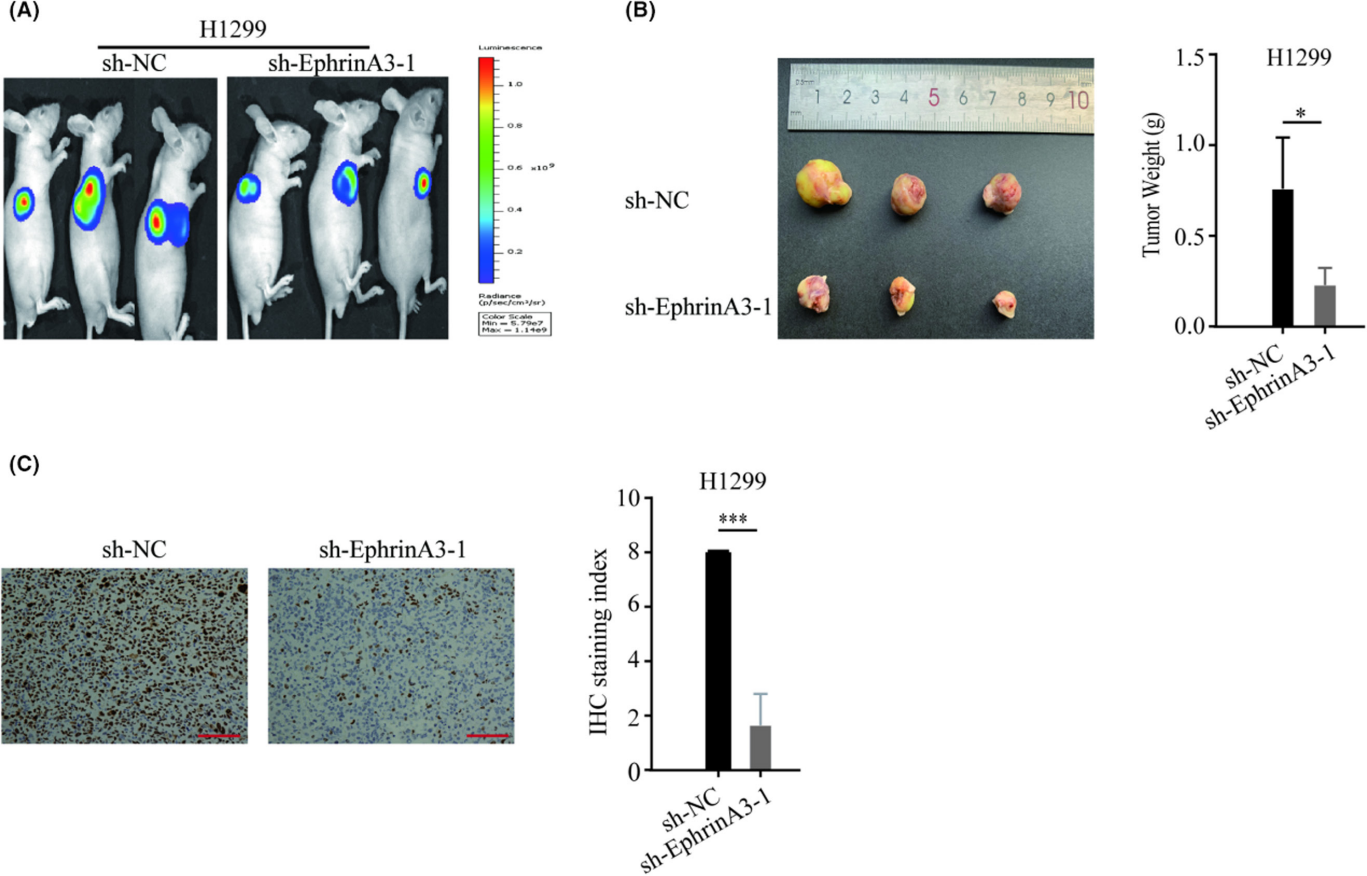
EphrinA3 potentiates LUAD growth in a murine xenograft model. (A–C) Bioluminescence imaging and immunohistochemical assay showed that EphrinA3 knockdown inhibits tumor growth in vivo. Scale bar represents 100 μm; Data are mean ± SD. *p < 0.05, ***p < 0.001.

Finally, we examined whether EphrinA3 regulated the in vivo development of LUAD using a murine xenograft model. Bioluminescence imaging showed that the fluorescence intensity of tumors developed from EphrinA3 knockdown LUAD cells were remarkably lower than those from control cells (Figure 6A). In addition, the weight of subcutaneous tumors derived from EphrinA3 knockdown cells was also significantly lower than those from the parental cells (Figure 6B). Staining for Ki‐67 indicated that EphrinA3 knockdown reduced the proliferative rate of cells in xenograft tumor (Figure 6C). These results suggest that EphrinA3 knockdown inhibits LUAD growth in vivo.

## 4 DISCUSSION

EphrinA3 plays different roles dependent on the varied origins or types of cancer. It is oncogenic in the pathogenesis of various malignancies including liver cancer, and is tumor‐suppressive in ovarian serous adenocarcinoma, peripheral nerve sheath tumors, oral squamous cell carcinoma, and pancreatic ductal carcinoma. EphrinA3 can promote the metastasis of liver cancer, and the survival of pancreatic ductal carcinoma,^11^, ^12^, ^13^ whereas it inhibits the migration and angiogenesis of oral squamous cell carcinoma, and the survival, migration, and adhesion of peripheral nerve sheath tumor.^8^, ^9^, ^11^, ^12^ Nonetheless, TCGA and Kaplan–Meier database showed that EphrinA3 mRNA was expressed highly in LUAD than that in adjacent normal tissues, and contributed to poor prognosis in LUAD. In this study, we further verified the higher expression of EphrinA3 in LUAD compared to the adjacent tissues through immunohistochemistry, suggesting that EphrinA3 may be an oncogene in LUAD. Knockdown of EphrinA3 inhibited the proliferation and migration of LUAD cells and retarded in vivo tumor growth, while its overexpression promoted these malignant phenotypes. The discrepant roles EphrinA3 plays in different tumor is probably attributed to the distinct contexts of intracellular signaling networks. However, further studies are needed to explore whether EphrinA3 is differentially involved in lung cancer driven by different genetic mutations.

EphrinA3 evokes cell–cell signaling through binding to its cognate receptors including EphA1, EphA8, and EphA10. EphA1 was overexpressed in gastric cancer, and its expression level was significantly associated with poor prognosis.^14^ EphA1/A2 and EphrinA1 were implicated in tumor angiogenesis in the bladder.^15^ EphA8 acts as an oncogene and contributes to poor prognosis in gastric and breast cancer.^16^, ^17^ EphA10 was found to regulate EMT and in vitro sphere formation of oral squamous cell carcinoma cells, and promote the occurrence of pancreatic cancers.^18^, ^19^ TCGA database showed that EphA1 and EphA10 were expressed in LUAD, while EphA8 was almost undetectable. Here, EphA1 and EphA10 expression was confirmed by immunohistochemical assay and was found to co‐localize with EphrinA3 on LUAD cells. While these findings suggest that EphrinA3/EphA contributes to LUAD progression via direct neoplastic cell contact, it remains to be determined whether it exerts an oncogenic role on EphA‐positive (referred to as forward signaling) and/or EphrinA3‐expressing cells (reverse signaling).

In the mechanistic study, we found that EphrinA3 signals to activate PI3K/Akt and Ras/MAPK pathways and upregulate the cell cycle checkpoint protein cyclinD1, which might account for the pro‐mitotic role of EphrinA3. In addition, EphrinA3 elicits EMT of LUAD cells and upregulates MMP‐2/−9 responsible for extracellular matrix degradation, which are likely to underlie its effect on LUAD metastasis. Although the detailed mechanisms underlying EphrinA3‐induced EMT are still elusive, its crosstalk with TGF‐β pathway, a master regulator of EMT, is probably involved.^20^ Recent studies have revealed that soluble Ephrins/Ephs play a key role in the progression of various cancers.^21^ While it is unknown how EphrinA3 signaling upregulates MMPs, these extracellular proteases can also convert membrane‐anchored Ephrin/Eph into soluble isoforms,^22^ thereby forming a feedback regulatory circuit in carcinogenesis. Together, our findings demonstrate a critical role of EphrinA3/Eph signaling in the pathogenesis of LUAD, and suggest the applicability of EphrinA3 and downstream pathways in targeted treatment and prognostic evaluation of clinical LUAD.

## AUTHOR CONTRIBUTIONS

Ruzetuoheti Yiminniyaze, Xiujuan Zhang, and Ning Zhu drafted the manuscript, performed experiments, and analyzed the data. Jing Wang, Chengwei Li, and Gulinuer Wumaier participated in carrying out the experiment. Daibing Zhou, Jing Li, Jingwen Xia, Youzhi Zhang, Liang Dong, and Yuanyuan Zhang contributed to collection of clinical specimens. Shengqing Li designed this study and supervised the manuscript. All authors read and approved the final manuscript.

## FUNDING INFORMATION

This study was supported by the National Natural Science Foundation of China (Nos.81670045 and 81,970,048).

## CONFLICT OF INTEREST

The authors declare that the research was conducted in the absence of any commercial or financial relationships that could be construed as a potential conflict of interest.

## ETHICAL STATEMENT

The studies involving human participants were reviewed and approved by the Ethics committee of Huashan Hospital affiliated to Fudan University. All participants enrolled in the study have signed informed consent forms. The animal study was reviewed and approved by the Ethics committee of Huashan Hospital affiliated to Fudan University.

## Supporting information


Figure S1

Table S1

Table S2

## Data Availability

Several public databases were used for this study, such as cBioportal for Cancer Genomics (www.cbiopartal.org), The Cancer Genome Atlas (TCGA) (http://portal.gdc.cancer.gov), Gene Expression Profiling Interactive Analysis (GEPIA) (gepia.cancer‐pku.cn), and Kaplan–Meier Plotter (https://kmplot.com/analysis/).
