# Supplementary figures and images for: EphrinA3 is a key regulator of malignant behaviors and a potential prognostic factor in lung adenocarcinoma
Source: Cancer Med. 2022 Jun 30;12(2):1630–42. doi: 10.1002/cam4.4987 (PMC9883548; doi:10.1002/cam4.4987)

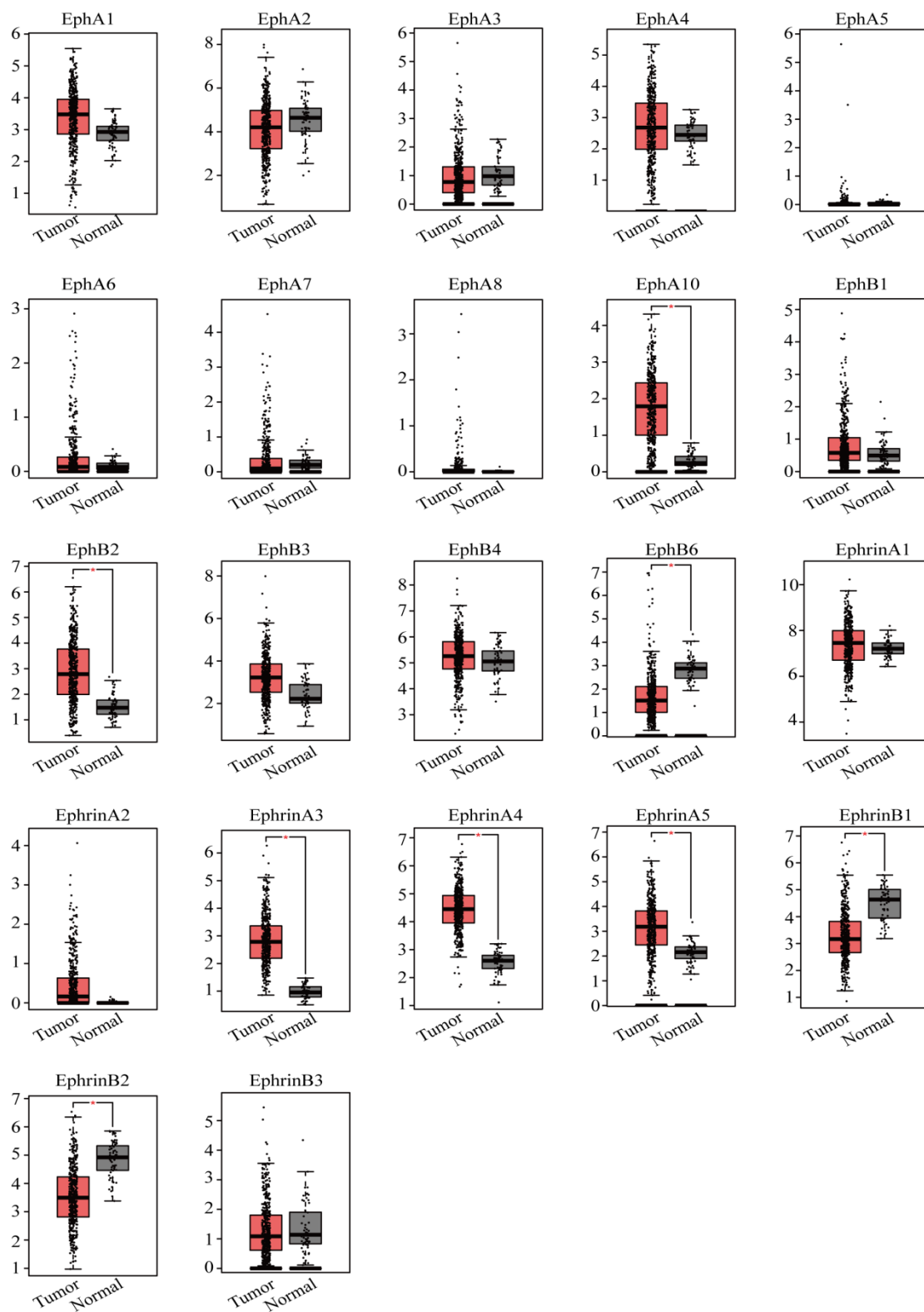

Supplement: Supplementary file 1 — Figure S1 [file CAM4-12-1630-s001.pdf]
