# Supplementary material for: EphrinA3 is a key regulator of malignant behaviors and a potential prognostic factor in lung adenocarcinoma
Source: Cancer Med. 2022 Jun 30;12(2):1630–42. doi: 10.1002/cam4.4987 (PMC9883548; doi:10.1002/cam4.4987)
Supplement: Supplementary file 2 — Table S1 [file CAM4-12-1630-s002.pdf]

Supplementary Table 1. Association of Ephs mRNA levels and overall survival in LUAD patients according to Kaplan-Meier Plotter database

	**HR[†] (95%CI[‡]）**	**Logrank *P***
**Receptors**		
EphA1	1.64(1.3-2.08)	$2.80E^{-05}$
EphA2	0.79(0.62-1)	0.053
EphA3	0.39(0.31-0.5）	$7.70E^{-15}$
EphA4	0.62(0.49-0.79)	$9.70E^{-05}$
EphA5	1.03(0.82-1.3)	0.79
EphA6	1.09(0.86-1.39)	0.48
EphA7	1.06(0.83-1.34)	0.66
EphA8	0.95(0.74-1.21)	0.66
EphA10	1.09(0.86-1.39)	0.47
EphB1	1.22(0.96-1.54)	0.097
EphB2	1.3(1.03-1.64)	0.026
EphB3	1.96(1.54-2.48)	$1.90E^{-08}$
EphB4	0.96(0.76-1.21)	0.7
EphB6	1.32(1.05-1.67)	0.018
**Ligands**		
EphrinA1	0.64（0.51-0.81）	$2.00E^{-04}$
EphrinA2	1.15(0.9-1.46)	0.26
EphrinA3	1.53(1.21-1.94)	0.00032
EphrinA4	1.15(0.91-1.45)	0.24
EphrinA5	1.05(0.84-1.33)	0.66
EphrinB1	1.49(1.18-1.88)	0.00079
EphrinB2	0.56(0.44-0.72)	$2.20E^{-06}$
EphrinB3	0.67(0.53-0.85)	$9.00E^{-04}$

[†]HR: hazard ratio; [‡]CI: confidence interval
