# Supplementary material for: EphrinA3 is a key regulator of malignant behaviors and a potential prognostic factor in lung adenocarcinoma
Source: Cancer Med. 2022 Jun 30;12(2):1630–42. doi: 10.1002/cam4.4987 (PMC9883548; doi:10.1002/cam4.4987)
Supplement: Supplementary file 3 — Table S2 [file CAM4-12-1630-s003.pdf]

Supplementary Table 2. Relationship between EphrinA3 expression level and clinicopathological characteristics in LUAD

Classification	Total	EphrinA3 expression		p
		High	Low	
**Sex**				
Male	28	11	17	0.077
Female	41	25	16	
**Age (years)**				
≤60	35	16	19	0.276
＞60	34	20	14	
**Grade**				
I-II	37	13	24	0.002
III-IV	32	23	9	
**TNM stage**				
I-II	44	17	27	0.003
III-IV	25	19	6	
